# Zinc uptake system ZnuACB is essential for maintaining pathogenic phenotype of F4ac^+^ enterotoxigenic *E. coli* (ETEC) under a zinc restricted environment

**DOI:** 10.1186/s13567-020-00854-1

**Published:** 2020-10-07

**Authors:** Guomei Quan, Pengpeng Xia, Siqi Lian, Yunping Wu, Guoqiang Zhu

**Affiliations:** 1 College of Veterinary Medicine (Institute of Comparative Medicine), Yangzhou University, 12 East Wenhui Road, Yangzhou, 225009 China; 2 Jiangsu Co-innovation Center for Prevention and Control of Important Animal Infectious Diseases and Zoonoses, Yangzhou, 225009 China; 3 Joint International Research Laboratory of Agriculture and Agri-Product Safety of Ministry of Education of China, Yangzhou University, Yangzhou, 225009 China

**Keywords:** Enterotoxigenic *E. coli* (ETEC), Zinc deficiency, ZnuACB, pathogenicity

## Abstract

Zinc is the second trace element of living organisms after iron. Given its crucial importance, mammalian hosts restrict the bioavailability of Zinc ions (Zn^2+^) to bacterial pathogens. As a countermeasure, pathogens utilize high affinity Zn^2+^ transporters, such as ZnuACB to compete with the host for zinc. It is essential for bacteria to maintain zinc homeostasis and thus maintain their physiology and pathogenesis. In an attempt to uncover the zinc transporter in F4^+^ enterotoxigenic *E. coli* (ETEC) C83902, we analyzed two RNA-seq data sets of bacteria samples when different zinc treatments (restriction or abundance) were applied. Considering data revealing that the high affinity zinc uptake system ZnuACB acts as the main transporter in ETEC C83902 to resist zinc deficiency, we deleted *znuACB* genes to study the role of them in ETEC C83902. The deletion of *znuACB* genes results in growth perturbation and a sharp decrease in the ability of biofilm formation and adhesion of bacteria in vitro. Taking the data together, this study demonstrates that the ZnuACB system is required for ETEC C83902 to acquire zinc, which highly contributes to ETEC pathogenicity as well.

## Introduction

F4^+^ enterotoxigenic *E. coli* (ETEC) is a kind of motile Gram-negative bacteria, consisting of three fimbrial variants F4ab, F4ac and F4ad. F4^+^ ETEC infection causes diarrhea of neonatal and post-weaned piglets in clinical practice, leading to serious economic losses in the swine industry [1, 2]. It is worth noting that diarrhea is commonly associated with insufficient dietary zinc (Zn^2+^) intake and ZnO supplementation in the feed was shown to efficiently reduce diarrhea [3].

Zinc is a second essential trace element in living organisms, including mammalians, bacteria, and plants, etc. It has different functions, such as a structural component, catalytic factor of molecules or proteins, and plays significant roles in cell growth, transcription, cell division, response to oxidative stress, apoptosis, and aging [4]. Eukaryotes exploit ZIP (Zinc import proteins) family protein, a membrane transporter family also known as SLC39, to transport zinc into the cell for its use by the cell and to restrict environmental zinc for pathogens to use [4, 5]. Apart from this, the host also requisitions the S100 family protein to sequester zinc locally in order to fight bacteria [6]. Interestingly, mammalian macrophages can also reorganize zinc concentration after phagocytosis, killing pathogens by zinc starvation or zinc toxicity [7, 8]. Actually, pathogens establish efficient systems to maintain zinc homeostasis to colonize host cells steadily.

**Figure 1:**
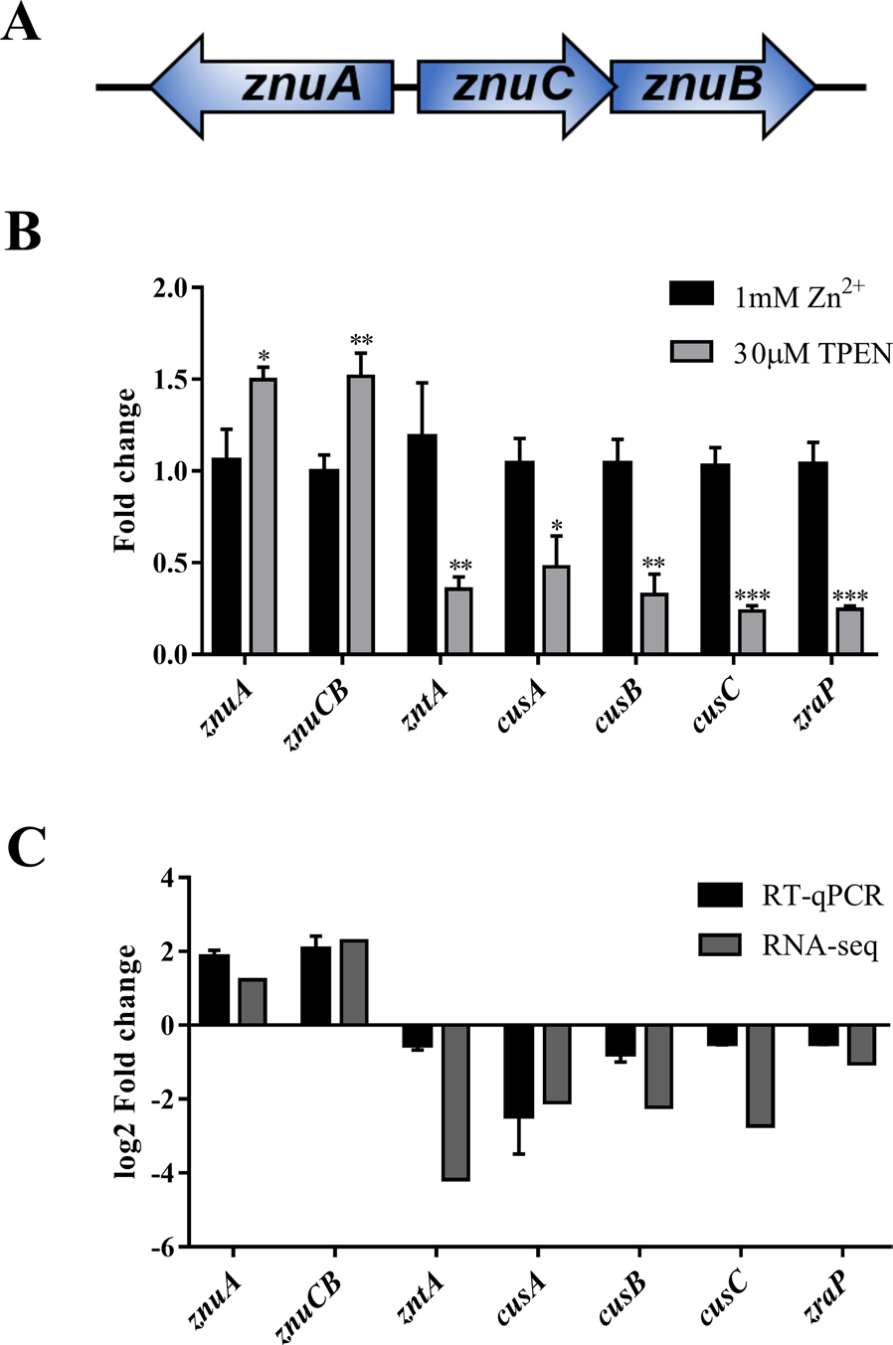
**Validation of expression profiling data by RT-qPCR. A** The diagram of the operon encoding ZnuACB transporter. **B** DEGs were validated by RT-qPCR. The relative gene expression level was calculated using the 2^−ΔΔCt^ value method and normalized by the *gapA* housekeeping gene. * significant at *p* < 0.05, ** significant at *p* < 0.01 and *** significant at *p* < 0.001. Data presented as mean ± standard deviations of three independent experiments. **C** Comparison of 7 DEG between RNA-Seq and RT-qPCR data. The y axes represent the log2 (fold change) measured by RNA-Seq and RT-qPCR, respectively.

For bacteria, the bioavailability of zinc is strictly controlled by zinc efflux and influx systems. In Gram-negative bacteria, zinc uptake is primarily mediated by ZupT (low-affinity Zn uptake system), which belongs to the ZIP (ZRT-, IRT-like protein) protein family under an adequate zinc environment. The high-affinity transporter of bacteria is sufficient to deal with the host metal-ion restriction strategy [9]. The ZnuACB (Zinc Uptake) transporter is one of the most widespread high-affinity zinc uptake systems, which is characterized as the ABC (ATP-binding cassette) transporter family member. The Znu uptake system is composed of three proteins: a periplasmic solute binding protein (SBP) encoded by *znuA*; a transmembrane spanning permease regarded as a channel and encoded by *znuB*; and a cytoplasmic ATPase function for energy supply encoded by *znuC* [4, 9] (Figure 1A). It was revealed that deletion of *the znuACB* gene affects bacterial growth ability under a zinc deficiency environment and influences their colonization and pathogenicity, such as *E. coli, Salmonella, Brucella, Vibrio cholerae and Campylobacte* [10–15].

In this study, we use RNA-sequencing technology to find differentially expressed genes under zinc abundance and zinc deficiency conditions. RNA-seq results show that the inner membrane zinc ACB transporter executes a higher expression level under a zinc deficient environment. So, we characterized the role of ZnuACB in F4ac^+^ ETEC in vitro and assessed the change of virulence related to the phenotype. We demonstrate that the ZnuACB system is required for F4ac^+^ ETEC growth, and is sufficient to maintain bacterial biofilm formation and adhesion to IPEC-J2 cells in vitro under zinc deficiency.

## Materials and methods

### Bacterial strains and cell culture conditions

Enterotoxigenic *E. coli* C83902 (O8:H19: F4ac^+^, LT^+^, STa^+^, STb^+^) strain [16] and the isogenic mutants C83902 Δ*znuA*, C83902 Δ*znuB*, C83902 Δ*znuC*, and C83902 Δ*znuACB* were routinely grown in LB broth or on LB agar plates at 37 °C. Antibiotics were used at the following concentration: 100 mg/L for ampicillin, 34 mg/L for chloramphenicol. Wild type (WT) strain and mutants were grown in LB medium with different concentrations of ZnSO_4_. Zinc restriction was accomplished by addition of TPEN, N, N, N’, N’-tetrakis (2-pyridylmethyl) ethylenediamine (Sigma, St. Louis, MO, USA), which were dissolved in DMSO (Dimethylsulfoxide). IPEC-J2 cells (Porcine neonatal jejunal epithelial cell line) [17] were cultured in DMEM media (Gibco, NY, USA), supplemented with 10% fetal bovine serum (FBS) (Gibco, NY, USA). The cells were maintained in 75 mL flasks (Corning, NY, USA) at 37 °C in a humidified incubator in an atmosphere of 6% CO_2_.

### RNA sequencing assay

Methods of sample collection: ETEC C83902 were cultured in LB medium with 1 mM ZnSO4 or pretreated with 30 µM TPEN until OD_600_ reached up to 1.0. Then 5 × 10^9^ CFU bacteria (triplicate biological replicates in each group) were gently washed three times by sterile ultra-pure water and collected by centrifugation at 4000 rpm, then re-suspended in TRIZOL reagent (Takara, Tokyo, Japan), immediately frozen in liquid nitrogen and stored at −80 °C. Samples were sent to the Vazyme (Nanjing, China) company and processed with the following procedures: I. RNA extraction; II. Total RNA sample detection, (1) the concentration and purity of RNA were detected by Nanodrop. (2) Qubit can accurately quantify the concentration of RNA. (3) Agilent 2100 accurately detects the integrity of RNA (RIN value); III. Library construction and library inspection: accurate quantification of effective concentration of the library by ABI Step One Plus Real-Time PCR system; IV. Sequencing: Illumina HiSeq sequencing is carried out after the quality control of the library is qualified; V. Data analysis: Gene expression is calculated by FPKM (Fragments Per Kilobase of exon model per Million mapped fragments), genes were considered differentially expressed between groups when the adjusted *p *< 0.05.

### Construction of isogenic target gene mutants for F4ac^+^ C83902 *E. coli*

Mutants were generated using the λ-Red recombinase method as described previously [16]. Briefly, sequences carrying the chloramphenicol resistance-encoding gene cassette derived from the template plasmid pKD3, which was amplified by PCR with specific primers, listed in Additional file 1, contain 5′ and 3′ regions of target genes of *znuA*, *znuB*, *znuC*, *znuACB*, and were transferred into C83902 containing plasmid pKD46 by electroporation. LB plates containing both chloramphenicol and ampicillin were used to screen the positive recombinant colonies. pCP20 plasmid, expressing Flp recombinase was used to eliminate the chloramphenicol cassette in the recombinants. The final mutant with target gene deletion from the C83902 strain was confirmed by combination of both PCR screening with specific primers and DNA sequencing.

### Measurement of bacteria growth under different zinc conditions

Zinc-depleted LB medium was pretreated with TPEN, N, N, N’, N’-tetrakis (2-pyridylmethyl) ethylenediamine (Sigma, St. Louis, MO, USA), to the indicated concentration and incubated for 2 h at room temperature before use. Overnight cultures of the wild type and different mutant strains were incubated 1:100 into LB medium, LB medium treated with TPEN, LB medium with or without ZnSO_4_ supplementation, respectively. The OD_600_ values were measured by spectrophotometer every hour.

### Biofilm formation assay

Biofilm formation assays were conducted as previously described [1]. For quantitative analysis of biofilm production, an overnight culture grown in LB medium (OD_600_ = 2.0) was diluted 1: 100 in biofilm-inducing medium (tryptone 10.0 g/L, yeast extract 5.0 g/L, NaCl 2.5 g/L, KH_2_PO_4_ 3.0 g/L, K_2_HPO_4_ 7.0 g/L, (NH_4_)_2_SO_4_ 2.0 g/L, FeSO_4_ 0.5 mg/L, MgSO_4_ 1.0 g/L, and thiamine hydrochloride 2.0 g/L). The new culture medium was added to 96-well microtiter plates (Corning, NY, USA), each well contained 150 μL of culture, and was incubated for 72 h at 30 °C without shaking. Then the culture was removed from the wells and gently rinsed three times with Milli-Q water. Bacterial biofilms were stained with Crystal violet (CV, 2%) solution for 15 min at room temperature. After incubation, the wells were rinsed with Milli-Q water three times and stained CV were dissolved with 95% ethanol. The spectrophotometer (BioTek, Winooski, USA) was used to measure the absorbance at 600 nm. Eight replicates were used in each experiment, and the whole experiments were carried out and repeated three times.

### Biofilm morphology

Morphology of WT and its Δ*znuACB* mutant biofilm in biofilm-inducing medium with or without TPEN were analyzed by scanning electron microscopy (SEM). Briefly, WT and Δ*znuACB* were incubated in 24-well tissue culture plates (Corning, NY, USA) with circular glass slides for 72 h, then the cells were fixed with 2.5% glutaraldehyde. The bacteria on slides were dehydrated with gradient alcohol, then prepared by critical point drying and sprayed gold. Then all samples were observed with SEM (Scanning Electron Microscopy) (GeminiSEM 300, Germany).

### Bacterial adherence assays

Binding specificity of the C83902 strain and different deletion mutants to IPEC-J2 cells were determined by adhesion assay [18]. Briefly, a monolayer of about 1 × 10^5^ IPEC-J2 cells were cultured in 96-well tissue culture plates (Corning, NY, USA). 1 × 10^7^ CFU of bacteria were collected and suspended in opti-MEM (Gibco, NY, USA), then added to the pre-prepared 96 well plate. After 1 h incubation, the cell-bacteria co-culture monolayer was gently washed three times with PBS and lysed with 0.5% Triton X-100 solution for 30 min. After lysis, the total cell-associated bacteria were diluted 1:100 in PBS, then spread on LB agar plates and incubated at 37 °C for the enumeration of adherent bacteria.

### RNA extraction and quantitative fluorescent PCR

Total RNA was extracted from bacterial samples using TRIzol reagent (Invitrogen, CA, USA). cDNA was synthesized using PrimeScript^®^RT reagent Kit with gDNA Eraser (Takara Bio, Tokyo, Japan). Quantitative real-time PCR was performed using AceQ^®^qPCR SYBR^®^ Green Master Mix (Vazyme, NJ, China). All the steps were carried out in accordance with the instructions. The primer sequence information is listed in Additional file 1. All data are presented after normalization to the *gapA* RNA expression observed in the same sample.

### Statistical analysis

All data were analyzed with GraphPad Prism Software (GraphPad Software, San Diego California USA) using the student t-test for independent samples. Three different levels of significance were defined: *p *< 0.05 was indicated by *, *p *< 0.01 was indicated by **, *p *< 0.001 was indicated by ***.

## Results

### The ZnuACB system contributes to zinc acquisition under zinc deficiency conditions

To identify differentially expressed genes (DEG) under zinc abundant and zinc restricted conditions, we used RNA-seq technology to detect the most upregulated and downregulated genes of ETEC C83902 in LB medium with 30 µM TPEN than those in the supplemented with 1 mM ZnSO_4_ LB medium. The results indicate that the sequencing reads were of good quality for expression analysis. The RNA sequencing read parameters are shown in the Supplementary section (Additional file 2). After analysis of differentially expressed genes (DEG), we identified 505 genes that were significantly upregulated and 523 genes that were significantly downregulated under zinc deficiency; an additional file with more detailed information of DEG is listed in Additional file 3. These DEG are involved in differently diverse cellular functions. We screened out zinc-related genes in these DEG (Table 1). Y75_RS09800 and Y75_RS09805 co-encode the high-affinity zinc uptake system ZnuACB transporter system mentioned above (Figure 1A). Y75_RS19925 encodes Zn^2+^/Cd^2+^/Pb^2+^/Hg^2+^ exporting P-type ATPase, ZntA, which belongs to the large and ubiquitous superfamily of ion and lipid transporters that hydrolyze ATP (adenosine triphosphate) to drive metal transport across biological membranes [19, 20]. Y75_RS02970, Y75_RS02965, and Y75_RS02955 encode cation efflux system proteins CusA, CusB, CusC respectively. CusA is a member of the resistance-nodulation-division (RND) protein superfamily. CusB belongs to the family of membrane fusion proteins and CusC is an outer membrane factor [21]. Y75_RS17075 encodes ZraP, a zinc resistance associated protein, which is the major Zn^2+^ containing soluble protein in Zn tolerance [22]. To validate the RNA-seq results, we performed the RT- qPCR assay to quantify the expression level of these screened genes (Figure 1B). The result confirmed that these gene are modulated by zinc insufficiency. Under zinc deficiency conditions, the expression of *znuACB* was significantly upregulated (*p *< 0.05) compared to zinc adequate medium and the expression levels of *zntA*, *CusA*, *CusB*, *CusC* and *zraP* were downregulated dramatically (*p *< 0.05), which shows a similar expression trend with that of the RNA-Seq data (Figure 1C).

**Table 1:** Differential expression of zinc-related genes in the zinc-restricted medium compared to zinc abundant medium

	Log2 fold change	p value	q value	Gene name or gene description
Upregulated genes				
Y75_RS09800	1.22902	0.0004	0.001993	*znuA*, zinc ACB transporter substrate-binding protein
Y75_RS09805	2.28633	0.00005	0.000301	*znuCB*, Mn^2+^/Zn^2+^ABC transporter ATP-binding protein
Downregulated genes				
Y75_RS19925	− 4.1821	0.00005	0.000301	*zntA*, Zn^2+^/Cd^2+^/Pb^2+^/Hg^2+^ exporting P-type ATPase
Y75_RS02970	− 2.09338	0.00005	0.000301	*cusA*, cation efflux system protein cusA
Y75_RS02965	− 2.21868	0.00325	0.012234	*cusB*, cation efflux system protein CusB
Y75_RS02955	− 2.72515	0.0009	0.004022	*cusC*, cation efflux system protein CusC
Y75_RS17075.	− 1.04709	0.01555	0.044091	*zraP*, zinc resistance-associated protein, zinc responsive, periplasmic protein with chaperone activity

log2 (fold change): log2(30 μM TPEN-FPKM/1 mM Zn^2+^-FPKM), *p*-value: *p* value of difference test, *q*-value: *p* value corrected by Benjamini–Hochberg correction method.

### Deletion of *znuACB* affects the growth ability of ETEC C83902 under zinc deficiency

**Figure 2:**
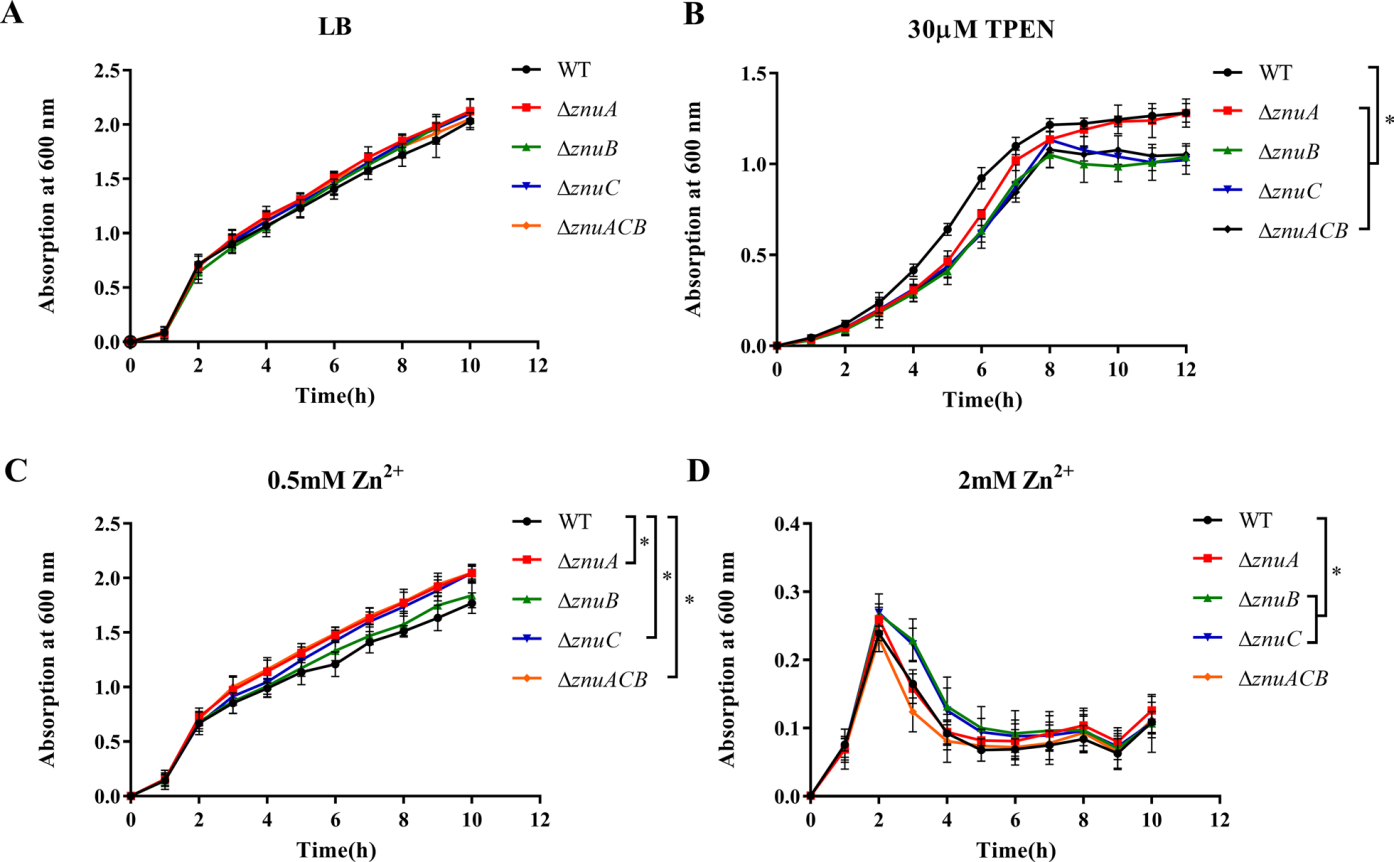
**Bacterial growth curves. A** WT strain and mutants were incubated in LB broth at 37 °C for 10 h with agitation, and OD_600_ optical was measured every 1 h. **B** WT strain and mutants were incubated in LB broth with 30 μM TPEN at 37 °C for 12 h with agitation. **C** and **D** WT strain and mutants were incubated in 30 μM TPEN pre-treated LB broth with 0.5 mM and 2 mM Zn^2+^ at 37 °C for 10 h with agitation, and OD_600_ optical was measured every hour. Data are the means and standard deviations from three independent experiments. *p < 0.05 (Student t test) compared to the corresponding wild type.

In view of RNA-seq results, we constructed mutants of the ZnuACB uptake system, namely C83902 Δ*znuA*, C83902 Δ*znuB*, C83902 Δ*znuC*, and C83902 Δ*znuACB*. The mutants were checked by both PCR and gene sequencing. The growth curves of mutants and WT strains in LB medium followed the same phases. However, after the early logarithmic growth period, all the mutants grew poorly compared to the WT strain when incubated in 30 μM TPEN pretreated LB medium (Figure 2). On the contrary, with the addition of 0.5 mM Zn^2+^ in the TPEN pretreated LB medium, all the mutants exhibited a growth advantage compared to the WT strain, whereas the growth curve in the TPEN pretreated LB with 2 mM Zn^2+^ showed zinc toxicity.

### Biofilm formation of *znuACB* mutants exhibited reduction features under a zinc shortage environment

**Figure 3:**
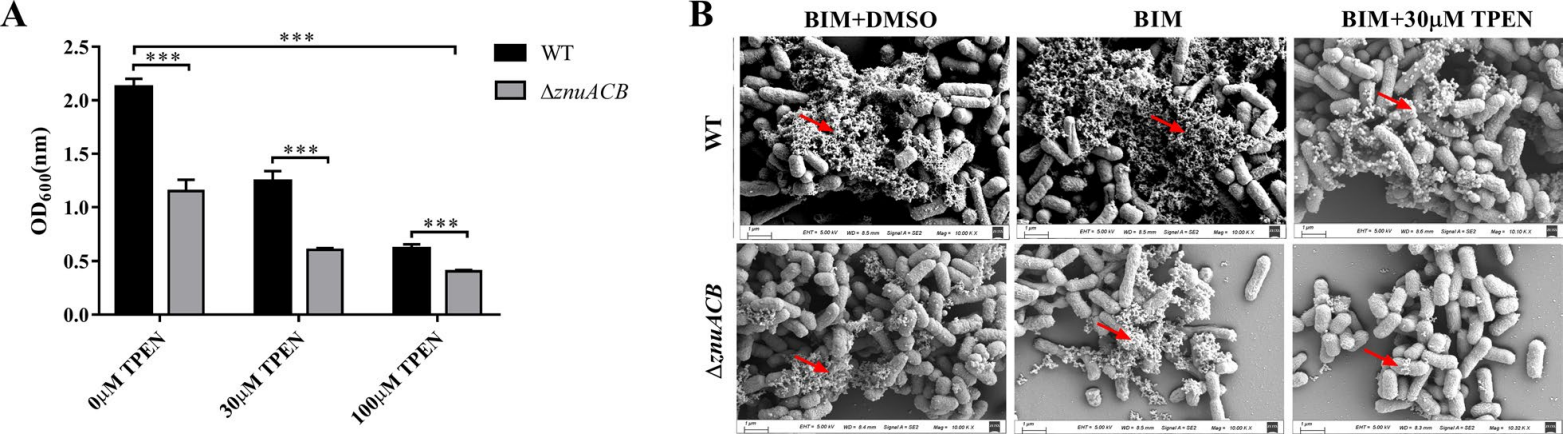
**Biofilm formation of WT strain and isogenic mutants of** ***znuACB*. A** Quantification of biofilm formation of WT C83902 and C83902 Δ*znuACB*. Surface-adhered biofilm on 96 well microtiter plates was quantified by measuring OD_600_ of ethanol-solubilized CV (2%) after biofilm staining. Data are shown as mean ± standard deviation of triplicate experiments. Significant differences between the mutant and WT C83902 are indicated by *p* < 0.001 ***. **B** The decrease of biofilm formation under zinc restriction was characterized by SEM. WT strain and C83902 Δ*znuACB* were fixed with 2.5% glutaraldehyde after incubation in BIM with or without 30 μM TPEN for 72 h. In order to eliminate the influence of DMSO, the TPEN solvent, BIM with DMSO was set as a control. Fixed bacteria were dehydrated by alcohol gradient, then dried by carbon dioxide critical point dryer and sprayed with gold, finally the sample biofilms were characterized by SEM. The biofilms are marked with a red arrow.

We compared the biofilm production of WT strain and mutants in biofilm-inducing medium (BIM) supplemented with or without zinc chelator TPEN. As the results show, we found that C83902 Δ*znuA*, C83902 Δ*znuB*, C83902 Δ*znuC*, and C83902 Δ*znuACB* mutants exhibit significantly decreased biofilm formation compared to WT (Figure 3A, Additional file 4). In BIM, the WT strain has an OD_600_ of 2.124 ± 0.08, while the C83902 Δ*znuACB* has an OD_600_ of 1.151 ± 0.10. With the addition of 30 μM of TPEN chelator in BIM, the WT strain exhibits a higher OD_600_ value (1.243 ± 0.09) than the C83902Δ*znuACB* (0.605 ± 0.02). In 100 μM TPEN chelated BIM, the WT strain displayed an OD_600_ of 0.616 ± 0.05, while the C83902 Δ*znuACB* mutant displayed an OD_600_ of 0.404 ± 0.01. With the addition of different concentrations of TPEN, both the WT strain and monogenic mutants appear to have a low biofilm productivity, however, the mutants still show lower production of biofilms than the wild type (Additional file 4). Then, SEM were performed to characterize the biofilm structure and morphology of the WT strain and C83902 Δ*znuACB* mutant. As shown in Figure 3B, the WT strain shows a sharp loss in biofilm production when exposed in the TPEN treated BIM compared to the BIM. When incubated in BIM, the C83902 Δ*znuACB* mutant exhibits obvious disruption of biofilm formation compared to the WT strain no matter whether it is with or without TPEN.

### Deletion of the ZnuACB system genes decreases the adhesion to IPEC-J2 cells under a zinc deficiency medium

**Figure 4:**
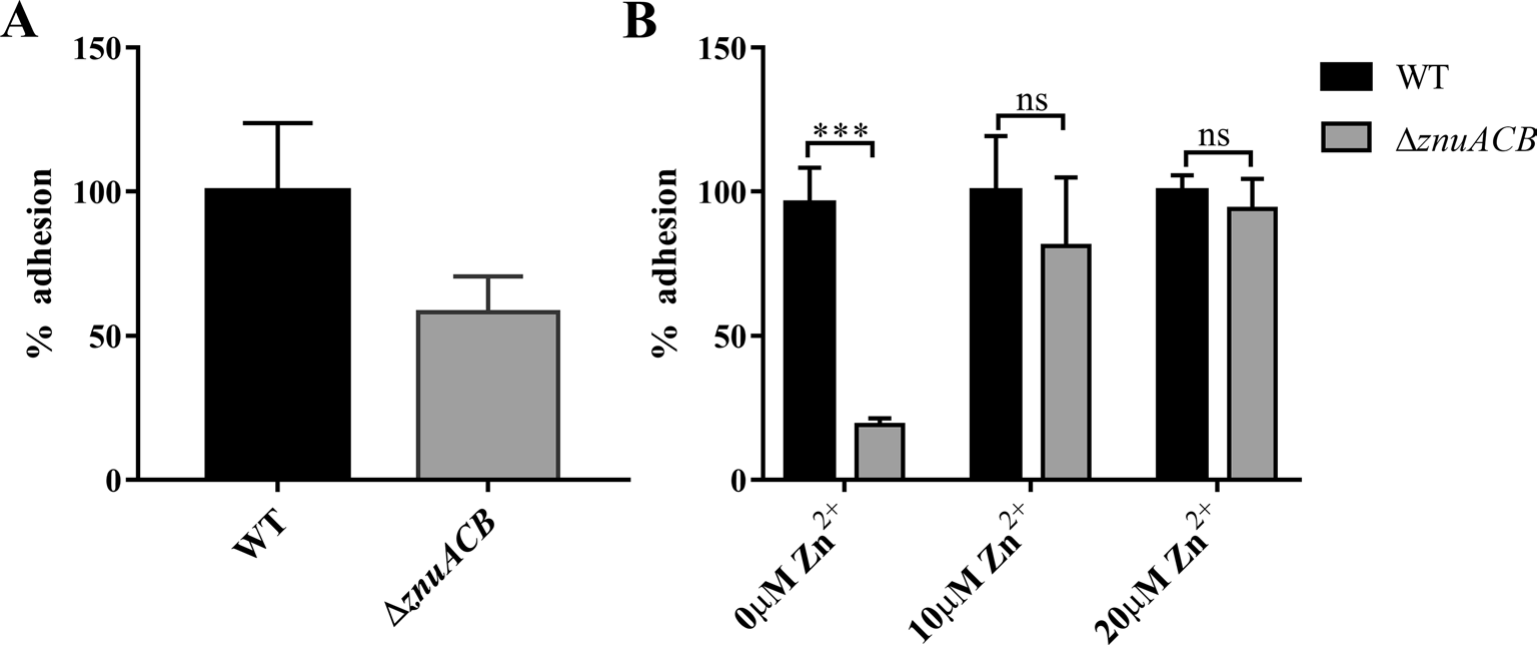
**Adherence of WT C83902 and C83902 Δ*znuACB***. **A** Deletion of C83902 *znuACB* decreases the adherence to IPEC-J2 cell. An adherence assay of WT C83902 and C83902 Δ*znuACB* to IPEC-J2 cell after pre-incubation in LB medium. **B** Zinc restriction led to a significant decrease of adhesion ability of C83902 Δ*znuACB*, while zinc supplementation can restore its adhesion ability. Bacteria were incubated in 30 μM TPEN pre-treated LB medium and supplemented with 10 μM or 20 μM Zn^2+^, then adherence assays of WT C83902 and C83902 Δ*znuACB* to IPEC-J2 cell were performed. The WT strain adhesion index was assumed to be 100%. Data are expressed as mean ± standard deviation of triplicate experiments. Statistically significant differences are indicated as ***p < 0.001 when compared to the WT strain.

For ETEC, the infection requires adhesion to host cells. In order to survey the role of a high ZnuACB affinity system during ETEC infection, an IPEC-J2 cell adhesion assay was performed. For statistical analysis, there was no significant difference in adhesion between the WT strain and mutants, whereas mutants exhibited a reduction of adhesion (C83902 Δ*znuA* 22.91%, C83902 Δ*znuB* 23.54%, C83902 Δ*znuC* 27.1%, C83902 Δ*znuACB* 42%, *p *> 0.05) under conventional LB medium to different degrees (Figure 4A, Additional file 5). We then conducted an adhesion assay under zinc deficiency conditions, utilizing a 30 μM TPEN chelated LB medium to culture bacteria, then co-incubated with IPEC-J2 cells. We observed that the C83902Δ*znuACB* mutant presents an 81% reduction of adhesion ability as compared to the WT strain (*p* < 0.001) (Figure 4B), but the other monogenic mutants still show non-significant decreased levels (C83902Δ*znuA* 18.64%, C83902Δ*znuB* 15.59%, C83902Δ*znuC* 28.14%, *p *> 0.05) (Additional file 5). We tested whether replenished zinc can remedy defection in adherence. For this purpose, we compared the adherence ability of the WT stain and C83902Δ*znuACB* mutant under 30 μM TPEN chelated medium with 10 μM and 20 μM Zn^2+^. As the amount of zinc is increased, the C83902 Δ*znuACB* mutant restored the ability of adherence to the IPEC-J2 cell, and reached a consistent level with the WT strain when 20 μM Zn^2+^ was added (Figure 4B).

## Discussion

Zinc, one of the transition metals, plays indispensable roles in both prokaryotic and eukaryotic cells at an appropriate concentration, which is controlled by precise mechanisms of host and pathogen cells. Since bacteria and hosts are aware of the significance of zinc, they have established a competitive relationship at the pathogen-host interface. Vertebrates employ multiple mechanisms to restrict zinc bioavailability to pathogens, which is generally termed “nutritional immunity” [9, 23, 24]. In particular, the host employs S100 family proteins, such as S100A7 protein psoriasis secreted by keratinocytes and S100A8/S100A9 dimer calprotectin secreted by neutrophil granulocytes to sequester zinc locally and extracellularly [4, 25]. ZIP family transporters usually maintain zinc levels intracellularly. In the host small intestine, absorption of zinc is achieved by high expression of ZIP14, which can keep the intestinal barrier intact in defense against bacteria [26]. ZIP4 also absorbs zinc from the small intestine and has roles in regulating intestinal epithelial cell function [27]. Additionally, ZIP7 is highly expressed in the crypts and is essential for intestinal epithelial cell turnover [28]. These strategies manipulated by the host not only meet the need of its own, but also protect the host from pathogen infection.

To overcome the competition, bacteria exploit multiple mechanisms to obtain zinc from the host. Studies have revealed that gram-negative bacteria have several zinc transporters to meet the requirement of zinc ions; bacterial infection subtly depends on their ability to import zinc [4, 29]. For most bacteria, a high affinity ZnuACB uptake system was recruited for bacteria to import zinc and against zinc deficiency, such as *E.coli*, *Salmonella*, *Brucella* etc. [30–32]. In addition to the ZnuACB permease, three putative transport systems that may facilitate translocation of Zn^2+^ across the inner membrane into the cell in *P. aeruginosa*: PA2911-PA2914, HmtA and PA4063-PA406. [33]. Interestingly, the mechanism of zinc acquisition is different between environmental and virulent species in *Francisella*. ZupT is the primary zinc acquisition transporter in environmental *Francisella*, however, it is not zupT but ZnuA that plays a crucial role in zinc uptake in virulent *Francisella* [34]. These data highlight the large and intricate arsenal of zinc import systems used by bacteria.

To elucidate the mechanisms used by ETEC C83902 to transport zinc, we used RNA-seq technology to seek how ETEC C83902 balance the zinc concentration under zinc adequate and deficient conditions in vitro. This led us to confirm that ETEC C83902 mainly uses the ZnuACB transporter to primarily mediate the importation of zinc. Simultaneously, to confront the zinc shortage, ETEC C83902 down-expressed (i) ZntA, Zn^2+^ exporting P-type ATPase; (ii) CusA, CusB and CusC, resistance-nodulation-division (RND) family proteins; iii) ZraP, Metallochaperones to reduce the zinc output. These three zinc export systems are essential to facilitate the translocation of zinc, and their expressions are highly decreased under zinc deficiency to ensure zinc accumulation in the cell [4]. These data indicate that ETEC C83902 “turn up” the zinc import system and “turn down” the zinc export transporters under zinc shortage to regulate the homeostasis of zinc ions.

In recent years, a number of studies have shown that the existence and integrity of the ZnuACB transport system can ensure bacteria survival, reproduction and virulence in a zinc-deficient environment [10, 11, 35]. But little is known about ZnuACB in ETEC, which especially causes piglet diarrhea that can be relieved by using zinc oxide as a feed additive. To clarify the function of ZnuACB in ETEC C83902, we constructed the Δ*znuA*, Δ*znuB,* Δ*znuC*, and Δ*znuACB* mutants. Most bacteria utilize the ZnuACB transporter to uptake zinc from the environment to ensure their growth. To investigate whether ZnuACB is involved in the zinc transporter in ETEC C83902, we examined the growth of mutants under TPEN pre-treated zinc deficiency medium. Expectedly, growth of the mutants in 30 μM TPEN pre-treated medium resulted in growth perturbation. We made a statistical analysis of the growth of different strains for each hour using the student t-test. In our experiment, all mutants had a growth disadvantage compared to the WT strain (*p *< 0.05) in a zinc restricted medium. But we noticed that C83902 Δ*znuA* exhibited the same growth trend as WT in the late stage of the growth curve, which is different than other mutants (Figure 2B), indicating that an additional zinc transporter system exists in C83902 to compensate for the role of ZnuA. We know that ZinT [36], an essential component of ZnuACB identified in *E. coli,* contributes to metal transfer to ZnuA, but whether the transport protein can compensate for ZnuA to deliver zinc still needs to be explored. To ensure that ZnuACB functions as a zinc importer, we added different concentrations of zinc back into TPEN pre-treated LB medium and found that zinc supplement restored the growth of the Δ*znuACB* mutants. All mutants show a growth advantage like the WT strain after 6 h of incubation in pre-treated medium supplemented with 0.5 mM Zn^2+^. Although statistical analysis shows that Δ*znuB* had no significant change, it still grew better than the WT strain (Figure 2C), whereas adding 2 mM Zn^2+^ led to growth restriction in all strains, and the result was likely to be an indicative of zinc toxicity caused by excessive zinc.

The ability of many pathogens to form biofilms is considered as an important factor to improve bacterial survival in the host and growth on contaminated abiotic surfaces. Thusitha et al. reported that deletion of *znuB* reduces UPEC CFT037 biofilm and swimming motility virulence phenotypes [11]. In accordance with the previous study, our investigation demonstrates that the ZnuACB system plays an important role in biofilm formation of ETEC C83902. Deletion components of ZnuACB transporter reduce the amounts of ETEC C83902 biofilm in BIM no matter whether with or without TPEN. With the addition of TPEN, both WT strain and mutants had a lowered biofilm biomass. SEM results are consistent with the biofilm quantitative results, the amount of biofilm formed by the Δ*znuACB* mutant was markedly less than the WT strain. However, the mechanism by which zinc shortage reduces biofilms in ETEC remains unknown. One possible explanation is that zinc deficiency downregulated the flagella phase-1 flagellin, which is reported to be related with the formation of biofilm in bacteria [12]. Our study also shows that the Δ*znuACB* strain had poor motility under zinc shortage in ETEC (data unpublished). But the mechanism still needs to be elucidated.

The first step in the pathogenesis of ETEC infection is bacteria attached to the host cells, allowing ETEC heat-labile (LT) and/or heat-stable (ST) toxin to be produced in close proximity to the intestinal epithelium. It has been demonstrated that the ZnuACB system is essential for the regulation of metal distribution in both the bacteria and the host, and necessary for adherence and colonization of gram-negative bacteria [11, 14, 37]. In our adhesion study, the results show that the Δ*znuACB* strain had an 81% reduction in adherence as compared to the WT strain in vitro after incubation in zinc deficiency conditions. This suggests that ZnuACB play a crucial role in ETEC C83902 adherence to IPEC-J2 cell under zinc deficiency. Based on our in vitro results, we can infer that the ZnuACB system contributes in colonizing ETEC C83902 in a zinc-limited gut. Our previous research has shown that FaeG, the major subunit of F4 fimbriae, can bind to the specific porcine aminopeptidase N (APN) receptor and tightly adheres to the porcine cell lines of the IPEC-J2 cell [38]. This means that FaeG is the most important and direct factor to evaluate the adherence ability for F4 *E. coli*. So, we detected *faeG* expression of *znuACB* deletion mutants by RT-q PCR method. As we expected, the expression of *faeG* in the Δ*znuACB* strain shows 0.4 folds (*p *< 0.01) reduction under the 30 μM TPEN treated zinc restricted environment (Additional file 6). The data show that the loss of *znuACB* is related to the lower expression of *faeG* with the decreased adhesion. Considering that the factors affecting bacterial adhesion are complicated, more work involved with Zinc regulation of the adhesin expression needs to be elucidated in the future.

Taken together, the results reported in this study provide evidence that the ZnuACB system is highly required by ETEC C83902 to conquer zinc deficiency, thus ensuring the formation of biofilm and the adherence to IPEC-J2 in vitro. These findings suggest that the reduction of the zinc importation ability results in loss of pathogenicity of strains and suggest the possibility of targeting zinc homeostasis in ETEC as a novel antimicrobial strategy.

## Supplementary information

**Additional file 1. Primes used in this study.**

**Additional file 2. Summary of RNA sequencing data from Z88 (1** **mM Zn**^**2+**^**) and Q88 (30** **μM TPEN) groups.**

**Additional file 3. Differentially expressed genes (DEG) of ETEC C83902 under zinc restricted (Q88) conditions compared to zinc abundant (Z88) conditions.** Locus: Genomic coordinates for easy browsing of the genes or transcripts, FPKM: Fragments Per Kilobase of exon model per Million mapped fragments, Q88_FPKM: The gene expression FPKM value of 30 μM TPEN condition, Z88_FPKM: The gene expression FPKM value of 1 mM Zn^2+^ condition, log2(Q88/Z88): log2(Q88-FPKM/Z88-FPKM), up-or-down: Whether the gene expression under the Q88 condition is up-regulated (up) or down-regulated (down) compared with Z88 condition, *p*-value: *p* value of difference test, *q*-value: *p* value corrected by Benjamini–Hochberg correction method, Description: Gene description information.

**Additional file 4. Biofilm formation of WT strain and mutants.** Quantification of biofilm formation of WT C83902, C83902 Δ*znuA*, C83902 Δ*znuB*, C83902 Δ*znuC*. Surface-adhered biofilm on 96 well microtiter plates was quantified by measuring OD_600_ of ethanol-solubilized CV (2%) after biofilm staining. Data are shown as mean ± standard deviation of triplicate experiments. Significant differences between the mutant and WT C83902 are indicated by *p* < 0.001 ***.

**Additional file 5. Adherence of WT C83902 and mutants.** An adherence assay of WT C83902 and C83902 Δ*znuA*,Δ*znuB* and Δ*znuC* to IPEC-J2 cells after pre-incubation in LB medium. Deletion of the C83902 *znuACB* component decreases the adherence to IPEC-J2 cells.

**Additional file 6. Expression level of fimbriae gene** ***faeG***. WT strain and Δ*znuACB* mutant were incubated in 30 μM TPEN pre-treated with LB medium. *gapA* was used as the normalizing internal standard. The transcriptional expression of the detected genes was measured by RT-qPCR. **indicates statistically significant difference when compared to the WT C83902 strain (*p* < 0.01).

## Data Availability

The datasets analyzed during the current study are available from the corresponding author on reasonable request.

## Abbreviations

ABC transporter: ATP-binding cassette transporter
BIM: biofilm-inducing medium
DEG: differentially expressed genes
DMEM: Dulbecco minimal Eagle medium
*E. coli*: *Escherichia coli*
ETEC: Enterotoxigenic *Escherichia coli*
FBS: fetal bovine serum
FPKM: fragments Per Kilobase of exon model per Million mapped fragments
IPEC-J2: porcine neonatal jejunal epithelial cell line
opti-MEM: opti-minimal essential medium
SEM: scanning electron microscopy
TPEN: N, N, N′, N′-tetrakis (2-pyridylmethyl) ethylenediamine
ZIP: Zinc import proteins
ZIP: ZRT-, IRT-like protein
