# Supplementary material for: Zinc uptake system ZnuACB is essential for maintaining pathogenic phenotype of F4ac+ enterotoxigenic E. coli (ETEC) under a zinc restricted environment
Source: Vet Res. 2020 Oct 7;51:127. doi: 10.1186/s13567-020-00854-1 (PMC7539401; doi:10.1186/s13567-020-00854-1)
Supplement: Supplementary file 1 — Additional file 1. Primes used in this study. [file 13567_2020_854_MOESM1_ESM.docx]

**Table S1 Primes used in this study**

| Primer | Sequence (5’-3’) |
| --- | --- |
| Δ*znuA*-Cm-F | TGCGACGCTCTCAACGACCGCTGGCCTGAACTGTGGCTCAGCAAAAACGCTGTGTAGGCTGGAGCTGCTTCG |
| Δ*znuA*-Cm-R | AATATCACACTTCTCATATTCATTACGATGATTGGTCGCATTATGTTACATATGAATATCCTCCTTAG |
| Δ*znuB*-Cm-F | TTATTATTTCCCGGTTGGTTAGCCGGGATCATGCTCGCCTGTGCCGCGTGTGTAGGCTGGAGCTGCTTCG |
| Δ*znuB*-Cm-R | ATCATACTGAGAATAAATAACAGTGCCGCACATAGCACCACCGACGGACATATGAATATCCTCCTTAG |
| Δ*znuC*-Cm-F | GATGAAGGGGTTATCAAGCGCAACGGAAAACTACGCATCGGCTATGTATGTGTAGGCTGGAGCTGCTTCG |
| Δ*znuC*-Cm-R | CCAAACATTGAAATAAACTCCGGATGCAGGGAAACAACTTCCGGTGTGCATATGAATATCCTCCTTAG |
| Δ*znuACB*-Cm-F | TGCGACGCTCTCAACGACCGCTGGCCTGAACTGTGGCTCAGCAAAAACGCTGTGTAGGCTGGAGCTGCTTCG |
| Δ*znuACB*-Cm-R | AATATCACACTTCTCATATTCATTACGATGATTGGTCGCATTATGTTACATATGAATATCCTCCTTAG |
| *gapA*-RT-F | CGTTAAAGGCGCTAACTTCG |
| *gapA*-RT-F | CGTTAAAGGCGCTAACTTCG |
| *znuA*-RT-F | TCGTTACCAACCTGCGTTTC |
| *znuA*-RT-R | ATGCATCTTTGGCTTTCCCC |
| *znuCB*-RT-F | GTCCTCTCTGATGTGTCGCT |
| *znuCB*-RT-R | CCGATGCGTAGTTTTCCGTT |
| *zntA*-RT-F | CTGACAGCACTTCCAACGTC |
| *zntA*-RT-R | CCCTGGCGATGTGATTGAAG |
| *zraP*-RT-F | CAGACAGCGTGGCAGAAAAT |
| *zraP*-RT-R | TCTCGTTTCACCCGTAACTCA |
| *cusA*-RT-F | ATCTCTCCGATGTGCAGGTT |
| *cusA*-RT-R | AGAGTCGCCAAACTGTGAGA |
| *cusB*-RT-F | GCTGACTTTTGCCCAGAGTT |
| *cusB*-RT-R | AGTCAGGAATGGTCAGGTCG |
| *cusC*-RT-F | AAGCGCAGACAGTAAACAGC |
| *cusC*-RT-R | CCGCTGGTCAGGCTTATAGA |
| *faeG*-RT-F | ACTCAGAAAACCTGATGGTGAAACT |
| *faeG*-RT-R | CCCCACCTCTCCCTAACACA |

Primers that contain Cm were used to amplify the sequences carrying the chloramphenicol resistance encoding gene cassette derived from the template plasmid pKD3.
