# Supplementary material for: Zinc uptake system ZnuACB is essential for maintaining pathogenic phenotype of F4ac+ enterotoxigenic E. coli (ETEC) under a zinc restricted environment
Source: Vet Res. 2020 Oct 7;51:127. doi: 10.1186/s13567-020-00854-1 (PMC7539401; doi:10.1186/s13567-020-00854-1)
Supplement: Supplementary file 2 — Additional file 2. Summary of RNA sequencing data from Z88 (1 mM Zn2+) and Q88 (30 μM TPEN) groups. [file 13567_2020_854_MOESM2_ESM.docx]

**Table S2. Summary of RNA sequencing data from Z88 (1mM Zn^2+^) and Q88(30μM TPEN) groups**

| Samples | Z8801 | Z8802 | Z8803 | Q8801 | Q8802 | Q8803 |
| --- | --- | --- | --- | --- | --- | --- |
| Total reads | 15758472 | 14472568 | 17296368 | 16701948 | 15533228 | 17403940 |
| Raw_reads | 15782160 | 14488852 | 17317482 | 16724066 | 15554072 | 17421758 |
| Clean_reads | 15758472 | 14472568 | 17296368 | 16701948 | 15533228 | 17403940 |
| Clean_bases | 2363770800 | 2170885200 | 2594455200 | 2505292200 | 2329984200 | 2610591000 |
| Raw_GC | 52.38% | 52.07% | 52.20% | 51.72% | 51.48% | 51.39% |
| Clean_GC | 52.38% | 52.07% | 52.20% | 51.72% | 51.48% | 51.39% |
| Raw_Q20 | 96.43% | 96.31% | 96.17% | 96.19% | 94.69% | 96.25% |
| Clean_Q20 | 96.43% | 96.31% | 96.16% | 96.19% | 94.68% | 96.25% |
| Raw_Q30 | 91.09% | 90.83% | 90.56% | 90.62% | 87.84% | 90.71% |
| Clean_Q30 | 91.08% | 90.83% | 90.56% | 90.62% | 87.83% | 90.71% |
| Total mapped reads | 12881227(81.74%) | 12410850(85.75%) | 14664831(84.79%) | 13340531(79.87%) | 12575079(80.96%) | 14154719(81.33%) |
| PE mapped reads | 12237948(77.66%) | 11863440(81.97%) | 13992580(80.90%) | 12592498(75.40%) | 11726664(75.49%) | 13439164(77.22%) |
|  | 643279(4.08%) | 547410(3.78%) | 672251(3.89%) | 748033(4.48%) | 848415(5.46%) | 715555(4.11%) |
| Uniq mapped reads | 9615534(61.02%) | 9358705(64.67%) | 11017616(63.70%) | 9778243(58.55%) | 8765989(56.43%) | 10446862(60.03%) |
| Multi mapped reads | 3265693(20.72%) | 3052145(21.09%) | 3647215(21.09%) | 3562288(21.33%) | 3809090(24.52%) | 3707857(21.30%) |

Total reads: the number of clean reads in sequencing data; Raw_reads: the number of reads of the original sequencing data; Clean_reads: the number of reads for high quality sequencing data; Clean_bases: Total number of bases of clean reads; Raw_GC :GC content of original sequencing data; Clean_GC: GC content of High quality sequencing data; Raw_Q20: The proportion of bases with a base mass value of not less than 20 in the original sequencing data; Clean_Q20: The proportion of bases with a base mass value of not less than 20 in high-quality sequencing data; Raw_Q30: The proportion of bases with a base mass value of not less than 30 in the original sequencing data; Clean_Q30: The proportion of bases with a base mass value of not less than 30 in high-quality sequencing data; Total mapped reads: The number and proportion of reads on the sequencing data can be compared by clean reads; PE mapped reads: The number and proportion of pairs of reads that can be compared in sequencing data clean reads; SE mapped reads: The quantity and proportion of sequencing data that can be compared by one end in clean reads; Uniq mapped reads: The number and proportion of unique positions in the genome that can be compared to the sequencing data clean reads; Multi mapped reads: The proportion of clean reads data that can be compared to multiple locations of the reference genome.
