# Supplementary figures and images for: Zinc uptake system ZnuACB is essential for maintaining pathogenic phenotype of F4ac+ enterotoxigenic E. coli (ETEC) under a zinc restricted environment
Source: Vet Res. 2020 Oct 7;51:127. doi: 10.1186/s13567-020-00854-1 (PMC7539401; doi:10.1186/s13567-020-00854-1)

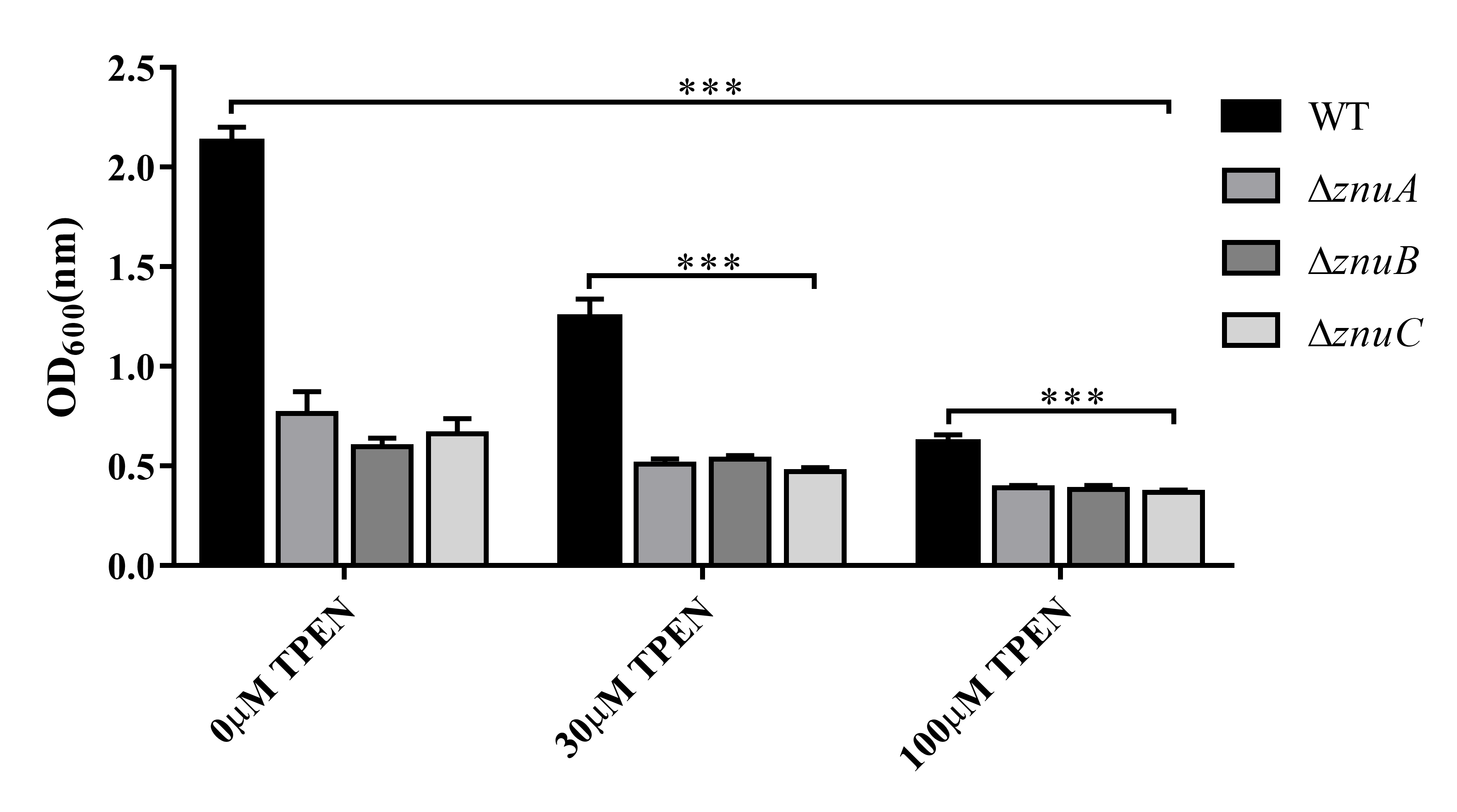

Supplement: Supplementary file 4 — Additional file 4. Biofilm formation of WT strain and mutants. Quantification of biofilm formation of WT C83902, C83902 ΔznuA, C83902 ΔznuB, C83902 ΔznuC. Surface-adhered biofilm on 96 well microtiter plates was quantified by measuring OD600 of ethanol-solubilized CV (2%) after biofilm staining. Data are shown as mean ± standard deviation of triplicate experiments. Significant differences between the mutant and WT C83902 are indicated by p < 0.001 ***. [file 13567_2020_854_MOESM4_ESM.tif]

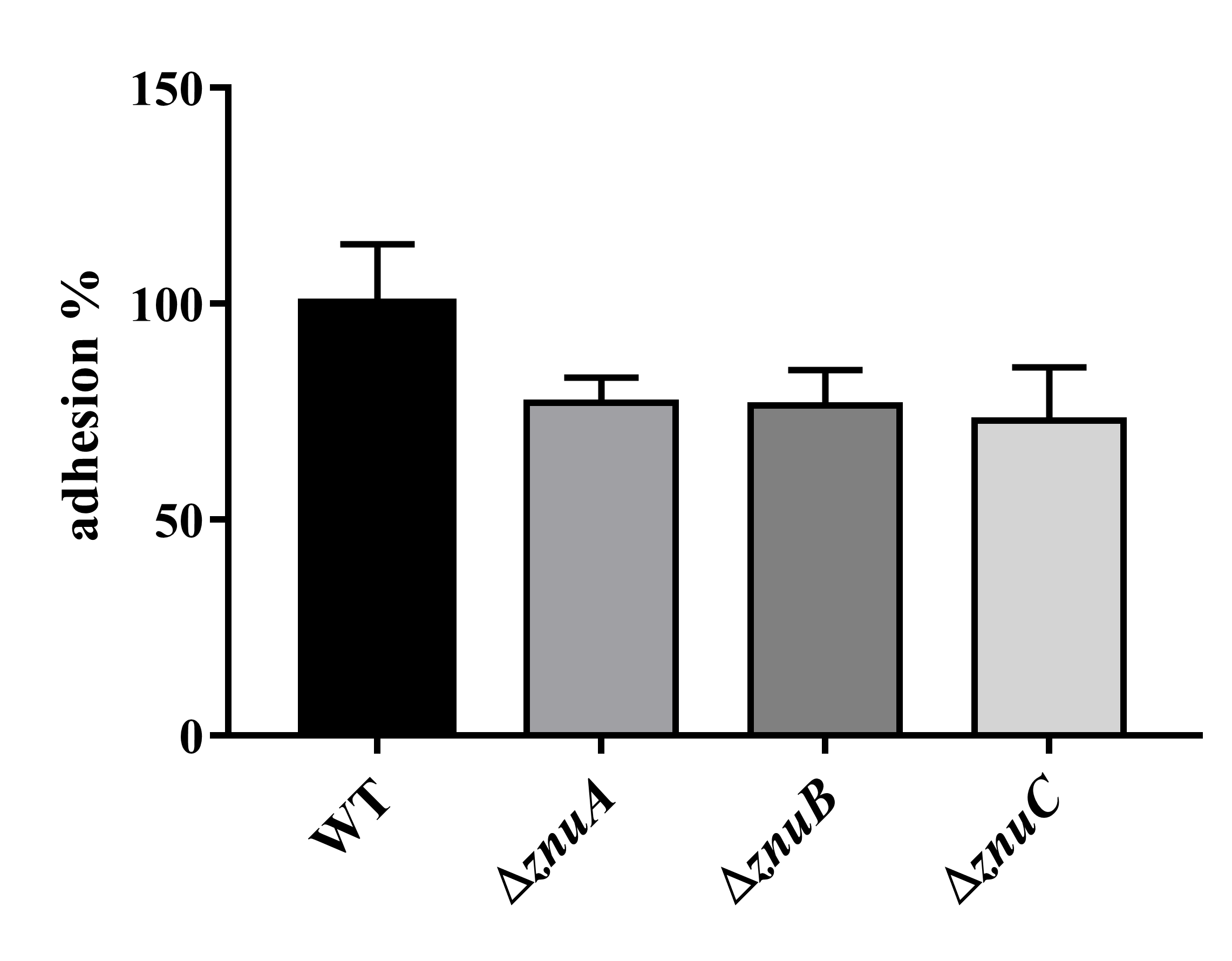

Supplement: Supplementary file 5 — Additional file 5. Adherence of WT C83902 and mutants. An adherence assay of WT C83902 and C83902 ΔznuA,ΔznuB and ΔznuC to IPEC-J2 cells after pre-incubation in LB medium. Deletion of the C83902 znuACB component decreases the adherence to IPEC-J2 cells. [file 13567_2020_854_MOESM5_ESM.tif]

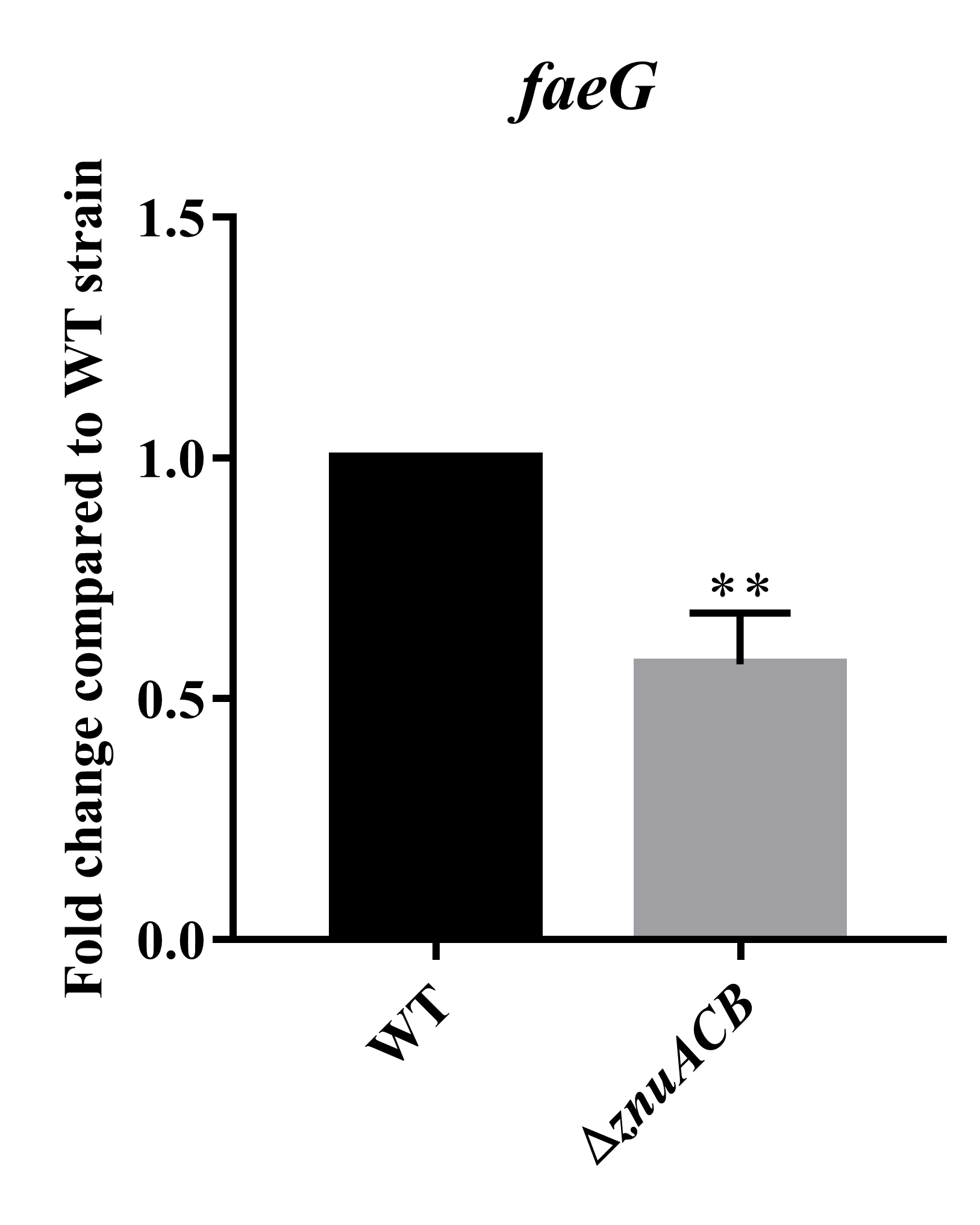

Supplement: Supplementary file 6 — Additional file 6. Expression level of fimbriae gene faeG. WT strain and ΔznuACB mutant were incubated in 30 μM TPEN pre-treated with LB medium. gapA was used as the normalizing internal standard. The transcriptional expression of the detected genes was measured by RT-qPCR. **indicates statistically significant difference when compared to the WT C83902 strain (p < 0.01). [file 13567_2020_854_MOESM6_ESM.tif]
